# Rewired lipid metabolism as an actionable vulnerability of aggressive colorectal carcinoma

**DOI:** 10.1080/23723556.2021.2024051

**Published:** 2022-01-27

**Authors:** Daria Capece, Guido Franzoso

**Affiliations:** a Department of Immunology and Inflammation, Imperial College London, London, UK; b Department of Biotechnological and Applied Clinical Sciences (DISCAB), University of L’Aquila, L’Aquila, Italy

## Abstract

Cancer cells reprogram lipid metabolism to fuel cell division, adaptation to stress, and metastatic dissemination. NF-κB transcription factors control this mechanism in aggressive Consensus Molecular Subtype (CMS)4 of colorectal carcinoma (CRC) via triacylglycerol (TAG) lipase, carboxylesterase 1 (CES1), thereby linking obesity-associated inflammation with metabolic adaptation and cytoprotection from lipid-induced toxicity. Our findings identify a potential therapeutic route to treat patients with metastasis-prone CRC and provide an example for targeting core tumor subtype-based vulnerabilities in cancers beyond CRC.

Obesity increases both the risk and mortality rate of colorectal carcinoma(CRC).^1^ Similar epidemiological associations with obesity have been reported in other types of human cancer, including breast, prostate, ovarian, and pancreatic carcinoma, underscoring the importance of lipid metabolism in oncogenesis.^2^ However, the incomplete understanding of how obesity influences aggressive tumor behavior has thus far limited the development of effective strategies to manage obese cancer patients.

While systemic alterations affecting hormone levels, insulin resistance, and gut microbiota may contribute to drive oncogenesis in obese patients, a prominent feature of CRC and other cancers in these patients is the preferential homing of tumors to the fat-rich visceral omentum or peri-glandular regions.^3,4^ Here, adipocytes and cancer cells establish a dynamic symbiotic relationship that profoundly influences tumor biology and clinical behavior. On the one hand, cancer-associated obese adipocytes (CAAs) release nutrients, growth factors, and inflammatory mediators that promote tumor growth, metastatic dissemination, and therapy resistance. On the other hand, cancer cells produce signaling molecules and chemokines that perpetuate inflammation and enhance lipolysis within CAAs As a result, cancer cells acquire adipocyte-like characteristics, including the ability to synthesize free fatty acids (FFAs) from endogenous carbon sources, such as glucose and glutamine, via *de novo* lipogenesis and accumulate excess lipids in the form of triacylglycerols (TAGs) and cholesteryl esters (CE) within lipid droplets (LD).^3,4^ Cancer cells also scavenge extracellular FFAs from circulating lipoproteins and neighboring CAAs via extracellular lipoprotein lipase (LPL) and FFA receptors, such as cluster of differentiation 36 (CD36; also known as fatty acid translocase) and fatty acid-binding proteins (FABPs).^2,4^

The extensive versatility of lipid metabolism confers on tumor cells the ability to adapt to their ever-changing microenvironment, particularly upon exposure to low nutrient and/or oxygen availability resulting from inadequate blood supply by the abnormal and insufficient tumor vasculature and increased metabolic demand of rapid tumor growth.^5,6^ Under these conditions, cancer cells enhance lipolysis to release endogenous FFAs stored in LDs and generate energy and reduced form of nicotinamide adenine dinucleotide phosphate (NADPH) via fatty acid oxidation (FAO).^2,6^ Increased FAO also protects tumor cells from anoikis by maintaining energy and redox homeostasis upon loss of attachment (LOA) to the extracellular matrix and migration to ectopic sites. During LOA, FAO-derived NADPH and adenosine triphosphate (ATP) are essential to counter oxidative and metabolic stress ensuing from decreased glucose uptake and flux through the pentose phosphate pathway (PPP).^6–8^ Tumor cell clones that are able to meet these challenges have a significant growth advantage and enhanced plasticity that allow them to outcompete other clones in metabolically diverse microenvironments, fueling tumor progression, disease recurrence, and metastasis formation. Beyond these roles under metabolic stress conditions, FAO provides cancer cells with a source of NAPHD and acetyl-CoA for membrane biogenesis and anabolic growth and an effective means of eliminating toxic lipids, which lead to reactive oxygen species (ROS) formation and ferroptosis, while contributing to the maintenance of cancer stem cells. Indeed, strong evidence indicates that increased FFA uptake and FAO flux are required for epithelial-to-mesenchymal transition (EMT), angiogenesis, tissue invasion, metastatic spread, and therapy resistance in many cancer types, including CRC.^6,7^

This dependence of tumor cells on FAO for cell division and adaptation to their microenvironment exposes a core vulnerability of metastasis-prone cancers and, in turn, an opportunity for therapeutic intervention. Numerous studies have established the potential clinical utility of targeting FAO in several cancer types, particularly those thriving in an adipocyte-rich microenvironment. For example, aggressive MYC proto-oncogene, bHLH transcription factor (*MYC*)^high^ triple-negative breast cancer (TNBC) critically rely on a high rate of FAO for energy provision, and pharmacologic or genetic inhibition of carnitine palmitoyl-transferase (CPT)1B or CPT2, the rate-limiting enzymes of FAO, impairs ATP production and tumor growth in *in vitro* cultures and patient-derived xenograft (PDX) models of *MYC*-overexpressing TNBC.^9^ Proximity to adipocytes also upregulates *CPT1A* expression and FAO flux in invasive CRC, enabling malignant cell survival in nutrient-deficient microenvironments, while *CPT1A* knockdown has been shown to abolish adipocyte-dependent protection, diminishing CRC organoid formation in 3D cultures and cancer growth in CRC xenograft models.^10^ Similar observations have been made in ovarian and prostate carcinoma.^4^

Another promising approach to blunt FAO in tumor cells is cutting off the supply of FFAs from neighboring CAAs. A high expression of FFA receptors, such as *CD36*, is characteristic of metastasis-initiating cells and portends poor clinical outcomes in many cancer types, including ovarian, prostate, and breast carcinoma and melanoma. Consistently, CD36 inhibition by genetic or pharmacologic means impairs tumor growth, cell migration, and metastasis formation in many of these cancers.^2,6^ CRC cells appear to rely on the FFA chaperones, FABP4 and FABP5, rather than CD36, for exogenous FFA uptake and lipid accumulation. Co-culture with adipocytes has been shown to increase *FABP4* expression and FFA content in CRC cells, resulting in enhanced ATP production, EMT, cell migration, and invasiveness via a mechanism that was reversed by pharmacologic FABP4 inhibition, while increased *FABP4* expression promoted metastasis formation in CRC xenograft models in mice.^11,12^ This FABP4/5-dependent mechanism of FFA uptake has also been described in ovarian, breast, and prostate carcinoma.^4,6,8^

Irrespective of their origin (whether they are derived from *de novo* lipogenesis or extracellular uptake), FFAs must be incorporated into LDs prior to being released by lipolysis for utilization via FAO or other metabolic pathways. Thus, an attractive strategy to halt FFA supply for FAO and, at the same time, trigger ROS formation and ferroptosis via polyunsaturated fatty acid (PUFA) accumulation would be to target non-redundant lipolytic enzymes in cancer cells.^4,13^ In support of this strategy, patatin-like phospholipase domain containing 2 (PNPLA2, best known as adipose triglyceride lipase or ATGL) has been shown to mediate a metabolic crosstalk between adipocytes and breast cancer cells that promotes tumor aggressiveness, EMT, and invasive potential in *ex vivo* co-culture systems.^14^ Similarly, increased expression of monoglyceride lipase (*MGLL*, best known as monoacylglycerol lipase or *MAGL*), which hydrolyzes monoacylglycerols into glycerol and FFA, increases the production of oncogenic signaling lipids and tumor aggressiveness in a wide range of cancers, including melanoma and ovarian and breast carcinoma.^6,8^ Accordingly, *PNPLA2* or *MGLL* inhibition by gene knockdown or small molecules has been shown to produce strong anti-tumor effects.^6^

While the potential efficacy of therapeutic strategies targeting lipid metabolism has been established in preclinical and clinical studies, translating these strategies into healthcare benefit has been complicated by their frequent toxicities, the heterogeneity of tumors, and contextual dependence of the lipidome on the tumor microenvironment. The development of genome-wide classification systems capable of distinguishing tumor subtypes with varying biological underpinnings and clinical behavior has recently provided a powerful new tool to help to untangle this complexity and contextually resolve tumor heterogeneity. In a recent study, we sought to take advantage of this approach to investigate whether and how obesity and altered lipid metabolism related to molecular subtypes of CRC and thereby identify core cancer-cell vulnerabilities amenable to therapeutic intervention. Using the Consensus Molecular Subtype (CMS) CRC classification,^15^ we discovered an intrinsic linkage of obesity with constitutive activation of NF-κB transcription factors, tumor-based inflammation, and fat catabolism in the mesenchymal CMS4 subtype, associated with stemness, stromal infiltration, EMT, and shorter overall survival (OS) and relapse-free survival (RFS) in CRC patients.^16^ By conducting a combined metabonomic and gene expression profiling, we identified carboxylesterase 1 (CES1) as an essential NF-κB-regulated TAG and CE lipase promoting tumor cell survival and metabolic adaptation in aggressive CRC.^16^ Increased *CES1* expression correlated with worse clinical outcomes in overweight, but not non-overweight CRC patients and was enriched downstream of NF-κB in CMS4 tumors, thus providing a mechanism for the aggressive clinical behavior of obesity-associated CRC and a potential therapeutic route that relates to the core biological underpinnings of tumor subtypes (Figure 1). Interestingly, *CES1* was alternatively upregulated by prognostically unfavorable amplifications of the gene encoding its transcriptional regulator, Hepatocyte nuclear factor 4 alpha (*HNF4A*), in the canonical CMS2 subtype,^16^ underscoring how different oncogenic pathways converge on CES1 to drive CRC (Figure 1). Notably, this tumor subtype-based distribution and unfavorable prognostic correlation distinguished CES1 from all other human intracellular TAG lipases,^16^ suggesting that CES1 plays a unique role in the etiopathogenesis of CRC.

**Figure 1.**
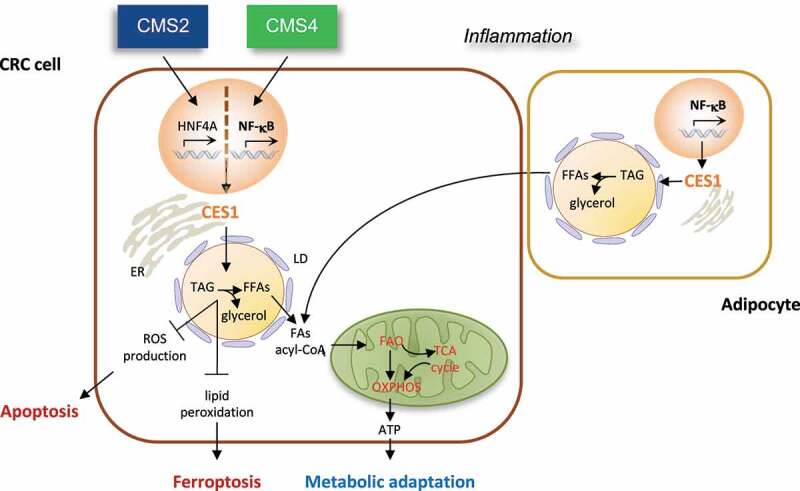
Carboxylesterase (CES)1-dependent metabolic rewiring in colorectal carcinoma (CRC) etiopathogenesis. CES1 is upregulated by HNF4A and NF-κB in the Consensus Molecular Subtype (CMS)2 and CMS4 CRC subtypes and promotes tumor cell survival by increasing triacylglycerol (TAG) breakdown to fuel fatty acid oxidation (FAO) and oxidative phosphorylation (OXPHOS) during starvation and preventing toxic lipid accumulation, which triggers apoptosis and ferroptosis. as CES1 is also expressed in adipocytes, we speculate it may also play a role in the symbiosis of CRC cells with adipocytes.

Mechanistically, we found that Ces1d/CES1 promotes CRC cell survival via at least two cell-autonomous mechanisms: 1) It increases TAG and CE breakdown to mobilize endogenous FFAs and produce ATP by FAO during starvation, thus enabling CRC cells to meet their energy demand; 2) it prevents the toxic buildup of neutral lipids that results in ROS production and phospholipid peroxidation, triggering apoptosis and ferroptosis^16^ (Figure 1). Accordingly, CES1 inhibition by knockdown or small molecules resulted in CRC cell death upon starvation *in vitro* and suppression of CRC growth in mouse xenograft models *in vivo*.^16^ Although our published data underscore the importance of the CES1-dependent metabolic mechanism in CRC cells, they do not exclude additional roles of CES1 in the symbiosis between adipocytes and cancer cells that fuel tumor progression and metastasis. Indeed, as *CES1* is also expressed in adipocytes,^17^ targeting lipolysis with CES1 inhibitors may cut off the FFA supply to cancer cells from both endogenous LDs and CAAs (Figure 1). As such, CES1 inhibition may be an effective means of suppressing EMT in inflamed CMS4 tumors and inducing anoikis upon LOA during the initial stages of metastasis. Future studies using single-cell (sc)RNA sequencing and patient-derived organoid (PDO) 3D co-cultures may help to clarify these potential added roles of CES1 in non-cancerous cells of the tumor microenvironment.

The identification of actionable therapeutic targets associated with the core biology of molecular tumor subtypes is an area of major clinical interest, particularly in mesenchymal tumors, due to their higher risk of metastasis and resistance to therapy.^18^ Given its profound effect on CRC cell survival and tumor subtype-based mechanism of action, CES1 inhibition may be an effective strategy to treat patients with aggressive types of CRC, such as microsatellite stable/non-hypermutated CMS4 and CMS2 tumors, which do not generally respond to immune-checkpoint immunotherapy.^18^ CES1-targeting agents may also be tolerated *in vivo*, considering the contextual specificity of CES1-dependent metabolic function for energy stress conditions and apparent lack of adverse effects of systemic CES1 blockade by knockout or small molecules in mouse models. While these findings support the potential clinical utility of more bioavailable CES1 inhibitors in CRC, it is unlikely that a CES1-targeting monotherapy can afford durable disease control, due to the eventual emergence of drug resistance. Previous studies have shown that targeting lipolysis or FAO can sensitize tumors to chemotherapy, radiotherapy, and anti-angiogenetic treatment, thus providing a rationale for combining CES1 inhibitors with standard of care agents.^6^ Notwithstanding this potential for synthetic lethality, the effective management of high-risk groups of CRC patients may require rational drug combinations built on an improved stratification of CMS4 (and CMS2) tumors that target synergistic cancer-cell co-vulnerabilities. Given the essential role of FAO and lipid metabolism in oncogenesis, an attractive strategy may be to combine tumor subtype-based interventions simultaneously blocking multiple metabolic pathways converging on FAO and interrelated cellular networks that govern energy homeostasis, redox balance, lipid trafficking/desaturation, and compensatory signaling mechanisms. Combinatorial treatments targeting the lipidome in cells of the tumor microenvironment, such as CAAs, may increase therapeutic efficacy by blunting the extracellular FFA supply and, at the same time, circumvent the ability of cancer cells to develop drug resistance. While future studies will determine whether CES1 blockade can be developed into an effective treatment in obese CRC patients, our findings may serve as an example for developing tumor subtype-based interventions also in cancers beyond CRC.
